# Comprehension of confidence intervals - development and piloting of patient information materials for people with multiple sclerosis: qualitative study and pilot randomised controlled trial

**DOI:** 10.1186/s12911-016-0362-8

**Published:** 2016-09-20

**Authors:** Anne C. Rahn, Imke Backhus, Franz Fuest, Karin Riemann-Lorenz, Sascha Köpke, Adrianus van de Roemer, Ingrid Mühlhauser, Christoph Heesen

**Affiliations:** 1 Institute for Neuroimmunology and Multiple Sclerosis and Department of Neurology, University Medical Center Hamburg-Eppendorf, Hamburg, Germany; 2 Unit of Health Sciences and Education, University of Hamburg, Hamburg, Germany; 3 Nursing Research Unit, University of Lübeck, Lübeck, Germany; 4 Institut für Didaktik in der Medizin, Michelstadt, Germany

**Keywords:** Patient information, Multiple sclerosis, Confidence interval, Interview, Pilot randomised controlled trial

## Abstract

**Background:**

Presentation of confidence intervals alongside information about treatment effects can support informed treatment choices in people with multiple sclerosis.

We aimed to develop and pilot-test different written patient information materials explaining confidence intervals in people with relapsing-remitting multiple sclerosis. Further, a questionnaire on comprehension of confidence intervals was developed and piloted.

**Methods:**

We developed different patient information versions aiming to explain confidence intervals. We used an illustrative example to test three different approaches: (1) short version, (2) “average weight” version and (3) “worm prophylaxis” version. Interviews were conducted using think-aloud and teach-back approaches to test feasibility and analysed using qualitative content analysis. To assess comprehension of confidence intervals, a six-item multiple choice questionnaire was developed and tested in a pilot randomised controlled trial using the online survey software UNIPARK. Here, the average weight version (intervention group) was tested against a standard patient information version on confidence intervals (control group). People with multiple sclerosis were invited to take part using existing mailing-lists of people with multiple sclerosis in Germany and were randomised using the UNIPARK algorithm. Participants were blinded towards group allocation. Primary endpoint was comprehension of confidence intervals, assessed with the six-item multiple choice questionnaire with six points representing perfect knowledge.

**Results:**

Feasibility of the patient information versions was tested with 16 people with multiple sclerosis. For the pilot randomised controlled trial, 64 people with multiple sclerosis were randomised (intervention group: *n* = 36; control group: *n* = 28). More questions were answered correctly in the intervention group compared to the control group (mean 4.8 vs 3.8, mean difference 1.1 (95 % CI 0.42–1.69), *p* = 0.002). The questionnaire’s internal consistency was moderate (Cronbach's alpha = 0.56).

**Conclusions:**

The pilot-phase shows promising results concerning acceptability and feasibility. Pilot randomised controlled trial results indicate that the patient information is well understood and that knowledge gain on confidence intervals can be assessed with a set of six questions.

**Trial registration:**

German Clinical Trials Register: DRKS00008561. Registered 8th of June 2015.

**Electronic supplementary material:**

The online version of this article (doi:10.1186/s12911-016-0362-8) contains supplementary material, which is available to authorized users.

## Background

Without knowledge and correct interpretation of numerical information, informed decision-making is impeded. The way statistical information is presented and explained has a high impact on understanding and interpretation [1]. In addition to information on absolute and relative risk reduction, thoughtfully developed information on confidence intervals (CI) for comparing treatment effects of immunotherapy options may be useful for communicating with people with multiple sclerosis (PwMS).

To correctly interpret study results, patients need to understand that study findings are effect estimates generated in a limited sample, which is assumed to represent the total population [2]. CI provide information about how accurate estimates are and thus add important information about the uncertainty of point estimates [3]. Understanding the relevance of CI in addition to basic event rates and absolute risk reductions may support patients and clinicians when evaluating study results and making informed choices [3]. The current Cochrane Handbook recommends to communicate both relative and absolute measures of risk and CI, which should be displayed in a ‘Summary of findings’ table [4]. However, approaches to explain CI to patients and consumers are rare [5] and no systematic evaluation exists.

For PwMS informed decision-making on disease-modifying drugs is highly relevant for self-managing their lives with this chronic progressive disease. PwMS are confronted with different choices concerning disease-modifying drugs, which are only partially effective but also bear relevant risks [6]. Adherence rates to disease-modifying drugs are as low as 30 % [7] indicating deficits also in the decision-making process. Communicating uncertainties may be an important step towards a better patient-medical-professional communication to achieve informed choices to which patients adhere to. Recent work has shown that addressing uncertainties does not induce anxiety and fear, but increases involvement and even adherence to disease-modifying drugs in MS [8]. In order to make informed medical decisions, PwMS not only need information about treatment effects in numbers, such as absolute risk reductions, but also information on the certainty of these estimates from clinical studies.

Therefore, this study aims to develop and pilot-test patient information (PI) materials to explain CI to PwMS. As currently no validated questionnaire assessing knowledge on CI is available, we aimed to develop and pilot-test a multiple-choice questionnaire to assess comprehension of CI.

## Methods

### Study design

Different PI materials were developed and pilot-tested according to the Medical Research Council’s framework for developing and evaluating complex interventions [9].

### Development

A systematic literature search was performed to identify studies evaluating approaches to explain CI. In total three different versions of PI materials were developed to explain CI to PwMS. The recommendations concerning the construction of evidence-based PI were considered [10, 11]. Different approaches were applied to explain CI; using the illustrative example of an apple farmer in two PI versions.

### Feasibility/piloting

**Fig. 1:**
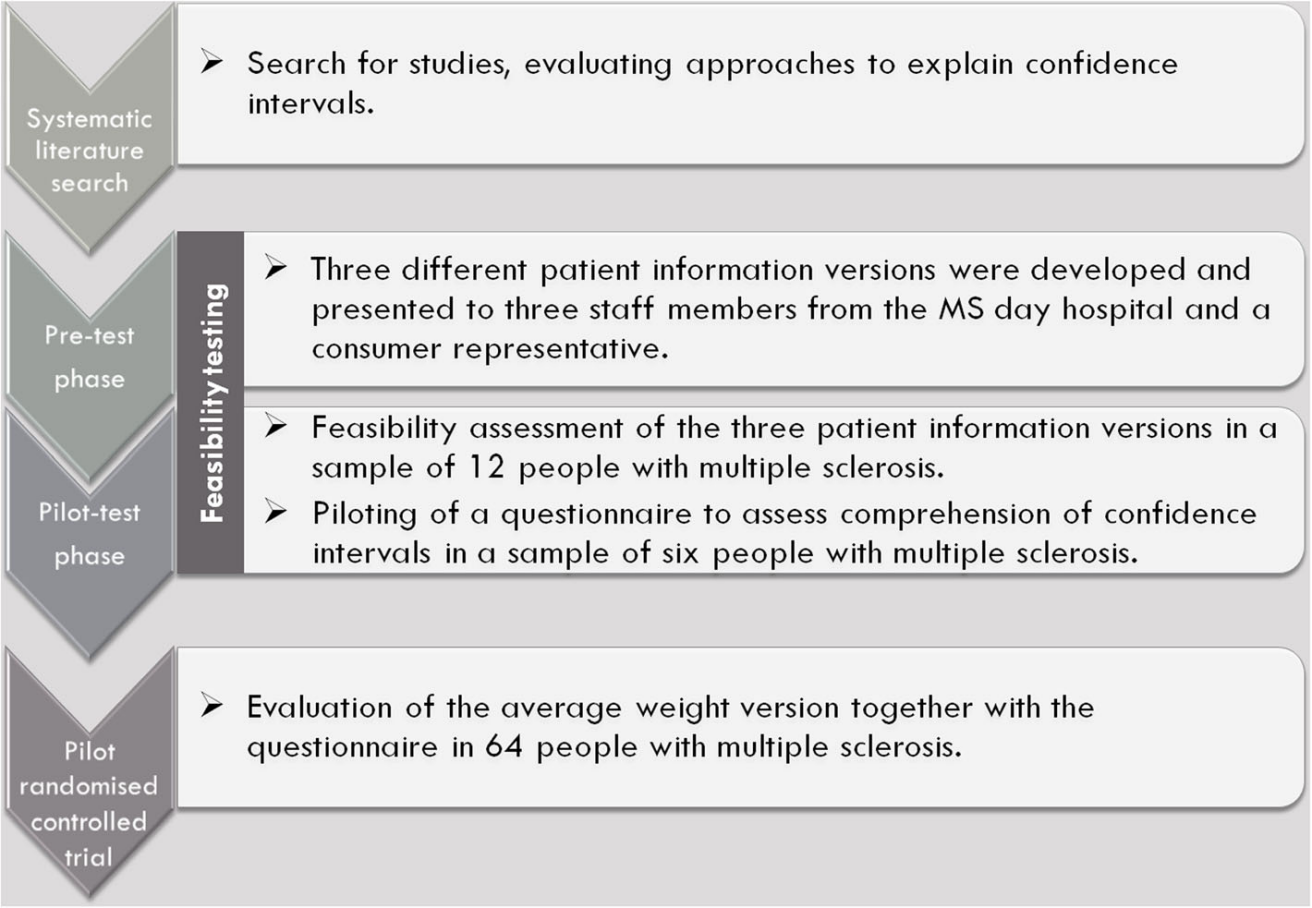
Study Flow

Assessment of feasibility included testing acceptability of PI materials and exploring to what extent the PI was judged suitable and attractive [12]. Practicability of the PI was tested by assessing the time needed to process the information, composition of text and graphic illustration as well as understandability. Feasibility of PI was tested in two consecutive stages. In a **pre-test phase,** three different PI versions were tested with non-academic staff members from the MS day hospital in Hamburg and a consumer representative from a self-help initiative. In a subsequent **pilot-test phase**, the three PI versions were piloted with a sample of PwMS. The multiple-choice questionnaire was tested with pilot-test phase participants [12]. Finally, in a **pilot-RCT,** one PI (average weight version, see below for details) was piloted together with the questionnaire in 64 PwMS (see Fig. 1).

### Participants

#### Pre-test and pilot-test phase

A convenience sample was used in the pre-test phase. In total three female staff members of the MS day clinic and one female consumer representative participated in the study.

In the pilot-test phase, a purposeful sampling strategy was applied to cover different distinct characteristics. In total 21 PwMS aged 18 years or older were selected from the MS day hospital, of whom eight declined to take part in the study due to timing issues. In total six of 13 PwMS received ≥ 12 years of education and thereof access to higher education Germany. Disease durations varied from 1 month to 19 years. Seven participants (54 %) were female. One patient dropped out at the beginning of the interview, because she expected a different input. Therefore, the final sample consisted of 12 PwMS.

#### Pilot RCT

Participants were recruited using mailing-lists of the MS day hospital, the local MS self-help society and other self-help initiatives [13–16].

After assessing the web-survey platform, participants were informed about the study and asked to provide demographic and disease specific data [17] and answer five questions on numeracy [18]. Participants were excluded with a notification by the system in case they filled in to be less than 18 years old or that they are not diagnosed with MS. After that, they were randomly allocated, using the UNIPARK randomisation sequence, to receive either the newly developed information or standard information. Directly after the intervention, they were asked to fill in the multiple-choice questionnaire.

### Setting and procedure

A think-aloud approach combined with semi-structured interviews was used to evaluate the PI and the questionnaire [19]. Participants (4 (staff members/consumer representative) and 12 (PwMS)) were asked to read the PI via a computer screen and verbalise their thoughts afterwards [19]. The teach-back method was employed to allow further improvement and clarification of the PI [20, 21].

All interviews, except one pre-test interview (telephone-interview), were held face-to-face and were audio-recorded at the MS day hospital by FF. There was no professional relationship between interviewer and participants. Interviews were not interrupted and recordings were of very good audio quality. Interviews ranged from 30 to 70 min.

The multiple-choice questionnaire with closed questions was developed following the recommendations by Haladyna et al. [22] and evaluated in the pilot-test phase and in the pilot-RCT. The average weight version on CI was tested against standard information on CI based on a formerly developed decision aid for PwMS [23] using the online survey software UNIPARK [24]. The average weight version, where a farmer wants to estimate the average weight of his apples, was chosen because this version was preferred by PwMS and contains all information considered to be important to understand confidence intervals (see 3.2.3 for details). The minimum sample size was set to 60 people, assuming that this would allow gaining sufficient information for the planned evaluation of the questionnaire and the PI in a larger sample. It was not aimed to reach a statistical significant difference between the two groups, yet to use the results after successfully piloting for the sample size calculation of a future RCT to evaluate the PI in a larger sample.

### Data analysis

#### Feasibility and pilot-phase

Interview recordings were transcribed using consistent rules [25] and transcripts were content analysed using Burnard’s approach [26]. The coding tree (Additional file 1) was developed along the gathered data and the structure of the interview guides. All transcripts were analysed using MAXQDA (version 11) and reviewed by a second person (AR).

#### Pilot-RCT

Data analysis was performed using the SPSS (version 21). Demographic data were analysed using descriptive statistics. An item analysis considering difficulty, distribution and discriminatory power was performed on the 6-items on CI comprehension [27]. Cronbach’s alpha (Kuder-Richardson) was calculated to determine internal consistency. Discriminant validity was assessed comparing the results to the abbreviated numeracy scale [18].

The questionnaire was complemented by four questions (Likert scale from 1–10) to evaluate an overall subjective rating of the understandability of the PI, the relevance of the topic, subjective knowledge and estimated subjective benefit of the PI.

## Results

### Systematic literature search

No study that explained CI to laypeople was identified (see Additional file 2 for detailed information).

### Feasibility and pilot-phase (written information)

#### Written patient information versions

**Fig. 2:**
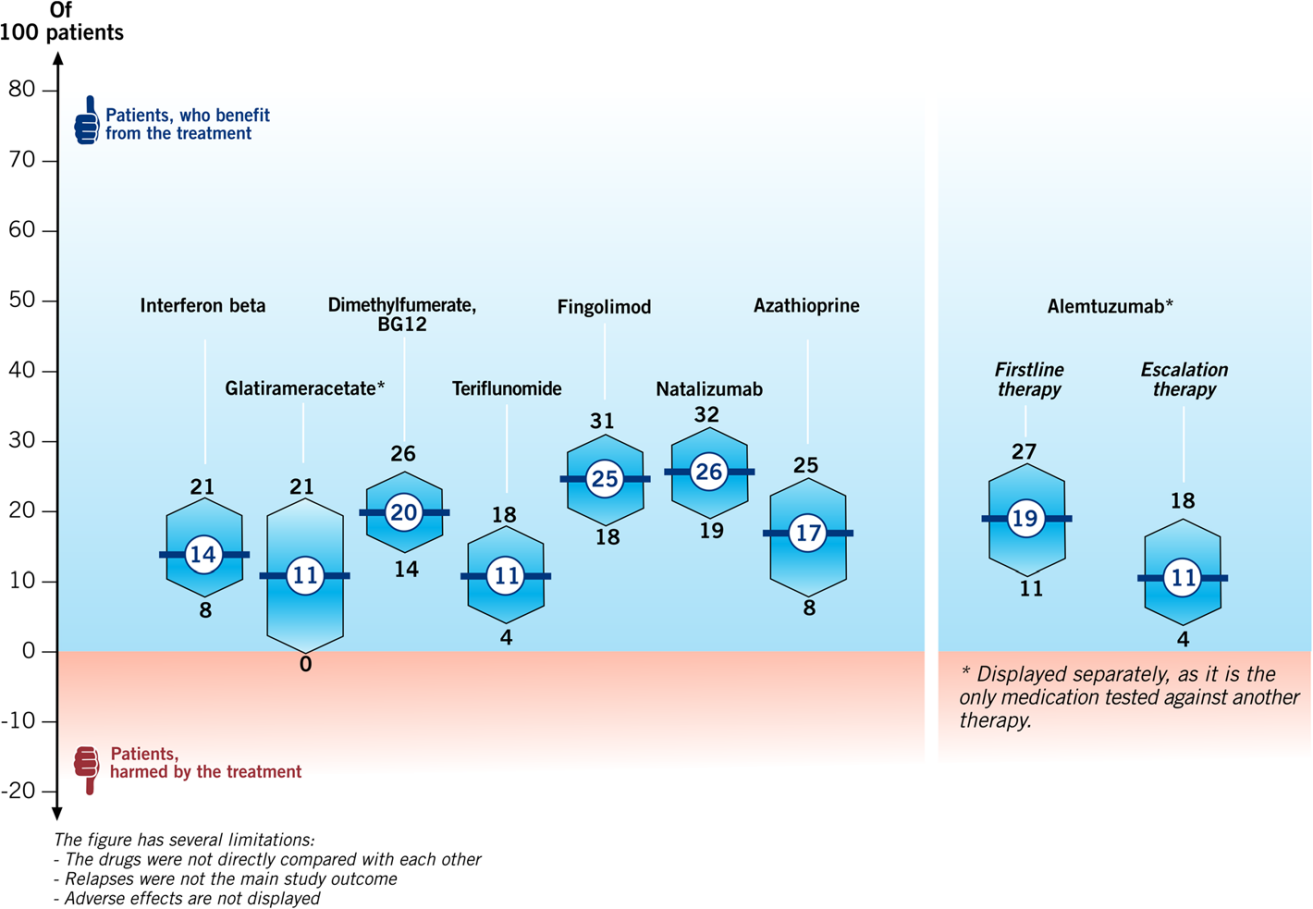
Confidence intervals (drug therapy effects in relapsing-remitting MS), Number of patients without relapses for 2 years due to drug therapy. References [30, 31, 35–39, 40–43]

A figure to display CI (Fig. 2) had been developed for an information platform on MS as part of the DECIMS (Decision Coaching in MS) project [28]. In the figure both the absolute risk reduction and CI are presented.

We decided to explain CI using a non-medical example followed by an MS specific example and developed three different PI on CI:

1. the average weight version
2. the worm prophylaxis version and
3. the short version.

Each version consists of an introduction, a main and a final part, with only the main part differing between versions. The introduction starts with a question from a virtual patient and is supposed to give participants an idea in which context and why CI are used. For the main part three versions were developed to cover different levels of complexity and different approaches to explain CI. The final part aims to transfer the gathered knowledge about CI to MS specific medications. While in the short version, CI are explained as briefly as possible without using an example, in the average weight and worm prophylaxis versions the story of an apple farmer is used to explain CI. In the average weight version, the farmer wants to estimate the average weight of his apples and CI are illustrated using small and large random samples of apples to estimate the average weight. In contrast, in the worm prophylaxis version, the farmer wants to test whether an anti-worm treatment is effective to prevent his apples from worm infestation. At first he tries to treat a small sample of apples, then a larger one, while he compares the results to untreated apples.

#### Pre-Test phase written patient information

During the pre-test the PI versions were revised before they were shown to the next participant. Significant changes were made in order to clarify contents. The narrative line was optimised and sentences were shortened. A statistician was introduced as a second virtual character, apart from the farmer, to better structure the information.

#### Pilot-test phase written patient information

For the pilot-test interviews, participants were first shown the average weight version, followed by the short and the worm prophylaxis version. We chose to present the short version between the other two versions to allow participants to rest between the two longer and more complex versions. In general, participants’ reactions ranged from positively interested on the one end, to being overwhelmed on the other (interview no. 8 and 11). In total four PwMS (interview no. 3, 5, 8 and 12) did comment on the need of explaining CI to patients. It was considered as important and PwMS wanted to read more about it, but there were also contrary voices (interview no. 5). Please see Additional file 3 for example quotes.

### Understandability

In total five PwMS (interview no. 1, 3, 6, 8, and 10) stated that the information on CI was easy to understand and one person that it was well described (interview no. 9). Other points, raised by one PwMS respectively, were: too many pages with same content making it difficult to stay attentive (interview no. 9); the information was partly confusing, a lot at once and some parts had to be read more than once (interview no. 11); and that some sections need shorter sentences to be better understood (interview no.10). No PwMS expressed that the content was not understandable.

In general, the presentation of numbers was described as a burden by four PwMS (interview no. 4, 5, 9 and 10). One PwMS reported that he found it difficult to tell whether numbers were derived from calculations of real figures or were made up as an example (interview no. 8). Two PwMS also stated that their numerical skills and their competencies in mathematics were weak (interview no. 4 and 8). On the contrary, another PwMS pointed out to remember the content visually presented, but later stressed to have problems with numbers (interview no. 9).

### Different versions and comparison of the different versions

In total six PwMS were positive about the apple farmer approach (interview no. 1, 3, 6, 8, 10 and 12). While five PwMS clearly expressed that they preferred the average weight version; three PwMS liked the worm prophylaxis version better and one PwMS liked the short version most. Another PwMS stated that he could not choose one, because every version yielded different information and only all three versions combined gave a complete picture of CI. Information about the favourite version was missing for two PwMS.

### Confidence intervals and multiple sclerosis specific medications

PwMS did not comment much on the final part of the PI. Two PwMS were pleased about the transfer to MS and MS medications (interview no. 4 and 8). Despite the dense and relatively difficult text, negative comments were rare (two persons, interview no. 5 and 6).

### Comprehension of confidence intervals

The comprehension of CI was mostly assessed by the teach-back phase and the multiple choice questionnaire. Questionnaire results are presented in section 3.3.

### Teach back

All PwMS of the pilot-test phase were asked to teach back the following aspects: definition of CI, benefits of using CI, width of CI, statistical significance and the apple farmer’s approach to answer his question (e.g. to estimate the average weight of his apples).

Overall, it was difficult for the PwMS to teach-back the content. However, some PwMS were able to teach-back the content quite well, whereas others could not teach-back the content predominantly correct. Some PwMS were able to teach-back the content of some parts while they had problems with other parts (see Additional file 4: Table S1).

#### Development and pilot-testing of the multiple choice questionnaire

The developed questionnaire initially consisted of eight multiple choice questions, of which four were visually illustrated. The questions addressed:

- the definition of CI
- the interpretation of CI and of point estimates based on an example
- the meaning of the width of CI and of the zero-line
- the interpretation of CI as well as influencing factors.

The questionnaire was pilot-tested with six of the 12 PwMS. Five of eight questions were answered correctly by five or more PwMS (see Additional file 4: Table S2).

### Further development of the multiple choice questionnaire

According to the feed-back of the PwMS, the questionnaire was further adapted. Two questions were deleted, as they addressed for the same content as other questions and wording of some questions was changed. The revised questionnaire was assessed again by four PwMS (see Additional file 5). No further need for revision was revealed.

### Pilot randomised controlled trial

**Fig. 3:**
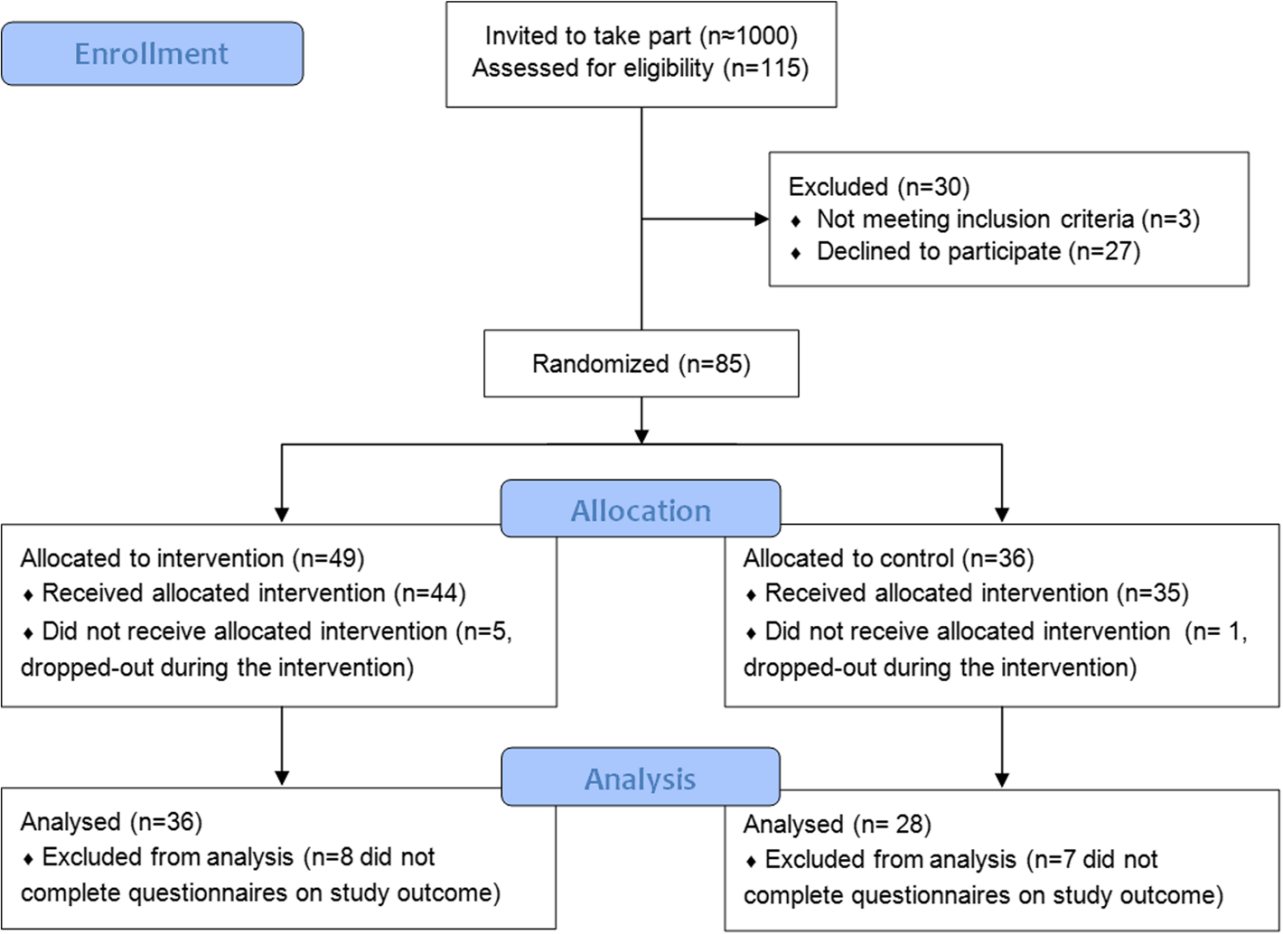
Flow diagram pilot RCT (CONSORT 2010) [44]

About 1000 persons were invited to take part via the mailing-lists. Participating PwMS were randomised to receive either the average weight version (IG) or standard information (CG). The survey was started by 115 PwMS, with 64 finishing the survey (36 IG/ 28 CG) (see Fig. 3).

Baseline demographics and disease specific data information are presented in Table 1. There were significantly more female PwMS in the CG. Otherwise there were no statistically significant differences in demographic parameters.

**Table 1:** Baseline data

Baseline data	IG *N* = 36	CG *N* = 28
Age (mean)	47.3	43.8
Females	19 (53 %)	22 (79 %)*
Education (highest degree)		
Secondary school	15 (41.7 %)	16 (57.1 %)
Academic degree	21 (58.3 %)	12 (42.9 %)
Disease course**		
CIS	0	2 (7.4 %)
RRMS	22 (61.1 %)	20 (71.4)
SPMS	9 (25 %)	4 (14.3 %)
PPMS	0	2 (7.1 %)
Other	3 (8.3 %)	0
Disease duration (mean)	9.1 years	9.5 years
Currently on Immunotherapy	18 (50 %)	11 (39.3 %)
PDDS (mean)	2.86	2.04

*IG* intervention group, *CG* control group, *CIS* clinically isolated syndrome, *RRMS* relapsing remitting multiple sclerosis, *SPMS* secondary progressive multiple sclerosis, *PPMS* primary progressive multiple sclerosis, *PDDS* patient determined disease steps

*Statistical significant difference (*p* = 0,039), **Missing data for two participants in the IG

PwMS in the IG answered 4.8 (mean, SD 1.3) of six questions correctly, while PwMS in the CG answered 3.8 (SD 1.2) questions correctly (mean difference 1.1 (95 % CI 0.42–1.69), *p* = 0.002, two-tailed t-test).

The questionnaire was developed to assess knowledge on CI in the context of study results on treatment options. As there was no comparative instrument available, the two groups were analysed separately concerning difficulty, internal consistency and discriminatory power [27].

The difficulty of the six items ranged between 0.43 and 0.94 in the IG and between 0.36 and 0.86 in the CG (Table 2).

**Table 2:** Item difficulty and discriminatory power

	Item 1	Item 2	Item 3	Item 4	Item 5	Item 6
Mean item difficulty (SD)						
IG (*N* = 36)	0.94 (0.23)	0.80 (0.40)	0.75 (0.44)	0.43 (0.51)	0.86 (0.35)	0.92 (0.28)
CG (*N* = 36)	0.68 (0.48)	0.86 (0.36)	0.54 (0.51)	0.36 (0.49)	0.5 (0.51)	0.82 (0.39)
Discriminatory power						
IG (*N* = 36)	0.17	0.33	0.41	0.45	0.27	0.23
CG (*N* = 36)	- 0.15	- 0.04	0.28	0.23	0.10	0.14

*IG* intervention group, *CG* control group

Cronbach’s alpha was 0.57 in the IG and 0.21 in the CG. Discriminatory power ranged between 0.17 and 0.45 in the IG and between 0.15 and 0.28 in the CG.

Due to a software error, only two of five questions on numeracy could be analysed. There was no significant correlation between numeracy and questionnaire results for the whole sample (0.161, *p* = 0.21). Numeracy in the CG correlated (Pearson’s r) positively (0.473, *p* = 0.01) with the mean sum score of the questionnaire, but not in the IG (-0.06, *p* = 0.7).

Concerning the general evaluation questions, the average weight version received better results. Results concerning understandability, subjective knowledge and benefits of the PI significantly favoured the IG (*p* = 0.01) (Table 3).

**Table 3:** Evaluation questions

Item	IG *N* = 36	CG *N* = 36
Understandability	6.5	4.5
Relevance	7.6	6.6
Subjective knowledge	6.6	4.8
Benefit of the PI	7.8	6.0

Understandability of the PI (1 = not understandable at all – 10 = very good to understand), Relevance of the topic CI (1 = not relevant at all – 10 = very relevant), Subjective knowledge on CI (1 = not understood at all – 10 = fully understood), Benefit of a PI on CI (1 = not helpful at all – 10 = very helpful)

## Discussion and conclusion

### Discussion

To our knowledge this is the first study to explain CI to patients. We developed and pre-tested three different PI versions on CI and piloted them successfully following the Medical Research Council’s guidance for developing and evaluating complex interventions [9]. Our pilot data indicate that CI can be made understandable through adequate PI interventions. PwMS contributed valuably to improve readability as well as understandability and enhanced comprehension. The majority of PwMS preferred either the average weight version or the worm prophylaxis version. The worm prophylaxis version was more difficult, but mirrored the setting of clinical trials very well, because of the treatment example. Therefore, this example could ease the transfer to immunotherapy decision making, as emphasised by some PwMS.

Statistical illiteracy by physicians and patients can result in misunderstanding study results, especially of numbers and verbal frequency statements [10, 29]. CI are beneficial for judging on the clinical relevance of statistical reporting and to reduce the chance of results being misinterpreted [3], because point estimates are complemented. Therefore, our graphical PI on CI, displaying both absolute risk reduction and significance of results, may be a step forward in patient education. The communication of CI could help to judge on the validity of the estimate by giving additional information to simply reporting point estimates. For example, the CI for the absolute risk reduction of glatiramer acetate (Copaxone®) concerning disability over 2 years ranges from zero to 21 and can be compared to other treatment options [30, 31]. However, not every patient needs to process and understand point estimates and CI as roles within decision making process have to be clarified [32] and thus might lead to a physician-led decision. Nonetheless, comprehensive information has to be made accessible in order to allow patients to get involved as much as they want based on the bioethical principle of autonomy [33]. Therefore, medical management should always strive for the highest possible degree of patient autonomy. This study is embedded in an ongoing project, in which a nurse-led decision-coaching intervention is evaluated to enable PwMS to make informed treatment choices [28]. The patient information will be made accessible on the online information platform after its evaluation in an RCT [34].

### Limitations of this study

There are some shortcomings of this study. PwMS of this pilot-study had the advantage of comparing all three versions with each other. The teach-back of the content indicated that some PwMS benefited from going through more than one version as they could teach back more information correctly after they had read the average weight and worm prophylaxis version. However, as the average weight version was always seen first by PwMS, the results might differ to another possible order. To account for this in a future RCT to evaluate all PI versions in larger sample [34], PwMS can watch a second video after having answered the questions. Due to the length and dense of information and drop-out rates it is not scheduled that PwMS see more than one PI material.

Caused by the small sample, the percentage of females in our pilot trial was imbalanced between the groups. However, we do not believe that this effected study results. Nevertheless, we will investigate on the impact of sex on the outcomes in the larger study.

Internal consistency and discriminatory power of the questionnaire were lower than aimed. For a high internal consistency, Cronbach’s alpha should have been over 0.70 and discriminatory power should have ranged between 0.40 and 0.70 [27], which was not reached for any question in the CG, whereas it was reached in two out of six questions in the IG. However, because the questionnaire consists of six questions only aiming to evaluate disease specific knowledge and comprehension on confidence intervals in general, high internal consistency would have been difficult to reach. Higher Cronbach’s alpha level in the IG indicates that gained knowledge leads to more consistent replies. The lack of a correlation of correct answers with numeracy in the IG might be due to the fact that a high score in numeracy is not necessarily helpful to understand the topic. However, this needs further evaluation.

With a mean difference of one question between groups clinical and practical relevance is an open question. Nevertheless, with more than two thirds of the questionnaire answered correctly by the IG it could be assumed that this kind of information on treatment options is understandable for PwMS. However, results need to be confirmed in a larger sample. Further, other presentation formats as for example videos might be a more attractive format for the user to receive information on CI than written information.

Finally, recruitment for the pilot-RCT was conducted via mailing-lists of the MS day hospital and self-help initiatives. Therefore, only PwMS, who are potentially interested in being updated by those institutions, were reached. Being aware that not all people read the newsletter, to us the response rate with 64 replies out of 115 who did login into the survey seemed sufficient for a pilot study and our recruitment target of 60 PwMS was fulfilled. However, a large study with a less biased sample is needed to evaluate the PI on CI.

### Conclusion

The pilot-phase shows promising results concerning acceptability and feasibility of different information materials on CI. PwMS may benefit from understanding CI, because they will be able to better compare different therapy options.

Understanding CI and other numerical data is of high importance for an informed treatment decision making process. Therefore, further research should focus on possibilities to explain numerical data of different formats in different patient groups.

## Additional files

Additional file 1: Coding tree. (DOC 30 kb) Additional file 2: Systematic literature search. (DOC 37 kb) Additional file 3: Example quotes patient information versions (pilot-phase). (DOC 33 kb) Additional file 4: Table S1. Teach-back results. **Table S2.** Results pilot-test questionnaire. (DOC 32 kb) Additional file 5: Multiple choice questionnaire “Comprehension of CI”. (DOC 192 kb)

## Abbreviations

CG: Control group
CI: Confidence interval
DECIMS: Decision coaching in multiple sclerosis
IG: Intervention group
MS: Multiple sclerosis
PI: Patient information
PwMS: People with multiple sclerosis
RCT: Randomised controlled trial
