# Supplementary material for: Comprehension of confidence intervals - development and piloting of patient information materials for people with multiple sclerosis: qualitative study and pilot randomised controlled trial
Source: BMC Med Inform Decis Mak. 2016 Sep 20;16:122. doi: 10.1186/s12911-016-0362-8 (PMC5029009; doi:10.1186/s12911-016-0362-8)
Supplement: Additional file 1: — Coding tree. (DOC 30 kb) [file 12911_2016_362_MOESM1_ESM.doc]

**Additional file 1: Coding tree**

| 1. **Feasibility**   Acceptability  Introduction  Narrative line  Practicality  Graphic illustration  Introduction  Average weight  Anti-worm treatment  Short version  MS specific ending  MS questionnaire  Understandability/ Clarity  Introduction  Average weight  Worm prophylaxis  Short version  MS specific ending  MC questionnaire  Other   1. **Comprehension**   Definition of confidence intervals  Application of using confidence intervals  Width of confidence intervals  Statistical significance and confidence intervals  True value  Sampling and estimation   1. **Methodology**   Think-aloud method  Teach-back method |
| --- |
