# Supplementary material for: Comprehension of confidence intervals - development and piloting of patient information materials for people with multiple sclerosis: qualitative study and pilot randomised controlled trial
Source: BMC Med Inform Decis Mak. 2016 Sep 20;16:122. doi: 10.1186/s12911-016-0362-8 (PMC5029009; doi:10.1186/s12911-016-0362-8)
Supplement: Additional file 2: — Systematic literature search. (DOC 37 kb) [file 12911_2016_362_MOESM2_ESM.doc]

**Additional file 2: Systematic literature search**

In order to describe the current state of literature, a research question using the PICO-principle was formulated to perform a search [1]. A systematic literature search was performed via OVID in MEDLINE, EMBASE and PsycInfo to identify studies in which interventions aiming to explain CI were applied. Further searches were conducted in DART Europe, Open Thesis, OPUS, ProQuest, ERIC, ESS, and Web of Science.

Here the term “confidence interval” and variations were combined with “comprehension” and related terms using the Boolean operator AND. The search resulted in 1293 hits (table B1). All titles were screened by one researcher (FF). Title screening was performed over inclusive to identify relevant literature. After this scan, 35 results remained and abstracts were scanned. The full-text of the remaining 10 publications was assessed by two researchers (FF and AR), using a pre-defined screening checklist. The checklist allowed structured screening by the inclusion criteria (explanation of CI or statistics to patients, explanation of CI to medical professionals, evaluation of comprehension of CI).

No studies were identified that explained CI to laypeople.

**Table: Results of the literature searches**

| **Databases** | **Results** |
| --- | --- |
| **MEDLINE, EMBASE, PsycInfo via OVID:** | 1272 |
| **Web of Science:** | 15 |
| **All other databases:** | 6 |

References

1. Higgins, Julian P. T. and Green, S., editor. Cochrane handbook for systematic reviews of interventions. Chichester: Wiley-Blackwell; 2011.
