# Supplementary material for: Comprehension of confidence intervals - development and piloting of patient information materials for people with multiple sclerosis: qualitative study and pilot randomised controlled trial
Source: BMC Med Inform Decis Mak. 2016 Sep 20;16:122. doi: 10.1186/s12911-016-0362-8 (PMC5029009; doi:10.1186/s12911-016-0362-8)
Supplement: Additional file 3: — Example quotes patient information versions (pilot-phase). (DOC 33 kb) [file 12911_2016_362_MOESM3_ESM.doc]

**Additional file 3: Example quotes patient information versions (pilot-phase)**

|  | **Participant quotes** |
| --- | --- |
| **General impression** | *“I really like it. It’s cheerfully designed, I find it very agreeable.”* (interview no. 8)  *“Oh ok, it’s a bit confusing. Maybe that’s because of me, but that is just so much information at once.”* (interview no. 11) |
| **Understandability** | [Question of the interviewer: “How would you assess the understandability overall?”]. *“It was good.” (interview no. 1)*  *That is clear so far, but is again so that I have problems in concentrating […] These are already three pages describing the same […] As a patient, I can’t pay attention this long anymore.” (interview no. 9)*  *“The font size was well chosen. I usually have difficulties reading, I skip a line very often, but here it was fine […] This kind of wording was beneficial. I didn’t have to read it twice.” (interview no.6)*  *“I didn’t pay attention. I have other things to worry about, which are more important to me.” (interview no. 5)*  *“This is where it’d be handy to be mathematician, maybe my kids would understand. But the majority of people, including me, don’t.(interview no. 4)* |
| **Different versions** | *“Very illustrative and well presented. The transfer to MS could be easily followed.” (interview no. 6)*  *“If somebody tried to explain this to me using fruits and apples, I would be very confused. I don’t see the relevance in it. You can’t compare it with diseases […] It’s silly! […] I found the apple example strange. Anti-worm treatment was very good.” (interview no. 4)* |
| **MS specific medications** | *“Ah, ok. Here we go. Now things become clear. This is also a question* [headline of the ending is: “How can this knowledge be transferred to MS drugs?”] *I’ve been asking myself.”(interview no. 8)*  *“This is much more meaningful than the things before. I simply understand it. There are 100 patients; one drug works better than the other. That’s a clear statement.” (interview no. 4)* |
