# Supplementary material for: Comprehension of confidence intervals - development and piloting of patient information materials for people with multiple sclerosis: qualitative study and pilot randomised controlled trial
Source: BMC Med Inform Decis Mak. 2016 Sep 20;16:122. doi: 10.1186/s12911-016-0362-8 (PMC5029009; doi:10.1186/s12911-016-0362-8)
Supplement: Additional file 4: Table S1. — Teach-back results. Table S2. Results pilot-test questionnaire. (DOC 32 kb) [file 12911_2016_362_MOESM4_ESM.doc]

**Additional file 4: Additional tables**

**Table S1**: Teach-back results

|  | **Good teach-back results** | **Mixed teach-back results** | **Not able to teach-back** |
| --- | --- | --- | --- |
| **Number of PwMS** | 3  (interview no. 1, 6 and 10) | 6  (interview no. 3, 4, 5, 8, 9 and 11). | 3  (interview no. 2, 7 and 12) |

PwMS = People with multiple sclerosis

**Table S2: Results pilot-test questionnaire**

|  | | **Question 1** | **Question 2** | **Question 3** | **Question 4** | **Question 5** | **Question 6** | **Question 7** | **Question 8** |
| --- | --- | --- | --- | --- | --- | --- | --- | --- | --- |
|  | **Number of correct answers** | 5 | 5 | 4 | 5 | 3 | 3 | 5 | 6 |

Number of correct answers according to questions from n=6 people with multiple sclerosis in the qualitative study
