# Supplementary material for: Comprehension of confidence intervals - development and piloting of patient information materials for people with multiple sclerosis: qualitative study and pilot randomised controlled trial
Source: BMC Med Inform Decis Mak. 2016 Sep 20;16:122. doi: 10.1186/s12911-016-0362-8 (PMC5029009; doi:10.1186/s12911-016-0362-8)
Supplement: Additional file 5: — Multiple choice questionnaire “Comprehension of CI”. (DOC 192 kb) [file 12911_2016_362_MOESM5_ESM.doc]

**Additional file 5: Multiple choice questionnaire “Comprehension of confidence intervals”**

Question 1

**What is meant by the term „confidence intervals”?**

*(Only one answer is correct)*

|  | They help to judge the certainty or uncertainty of study results. |
| --- | --- |
|  | They show the effectiveness of a drug. |
|  | They show in how many patients a treatment is effective and in how many it is harmful. |
|  | They are statistical aids, which have to be calculated by the reader at first. |

**
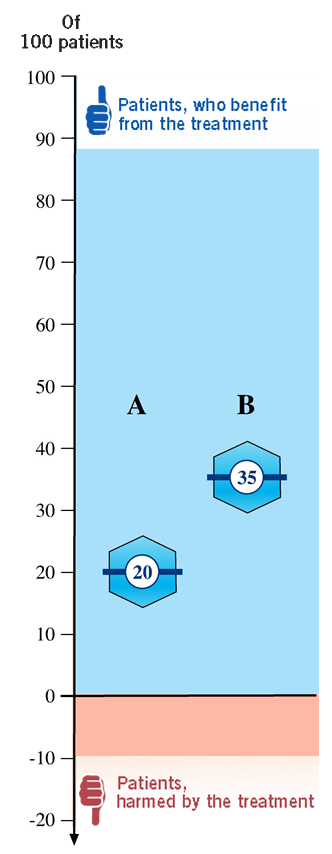
**

Question 2

**Here, treatment effects of two medications are graphically displayed.**

**Which medication is more effective?**

(Only one answer is correct)

|  | Medication A |
| --- | --- |
|  | Medication B |
|  | There is no difference between the medications |

**
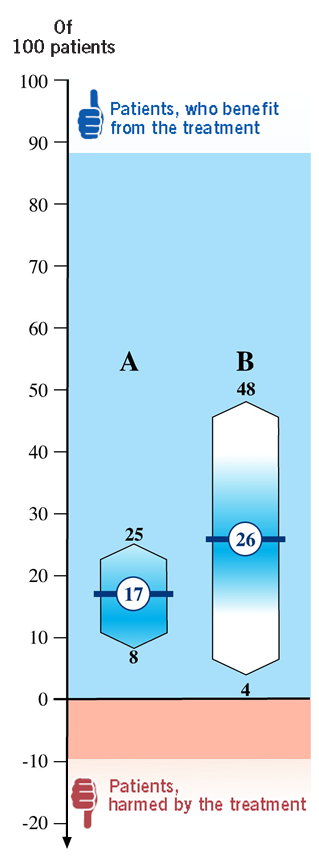
**

Question 3

**Here, treatment effects of two medications are graphically displayed. For which of the medications the benefit is demonstrated with more certainty?**

(Only one answer is correct)

|  | Medicament A |
| --- | --- |
|  | Medicament B |
|  | There is no difference between the medications |

Question 4

**What does it mean, when the confidence interval crosses the zero line, thus includes positive and negative numbers?**

(Only one answer is correct)

|  | It has no special meaning |
| --- | --- |
|  | Confidence intervals cannot reach into a negative area |
|  | The benefit of the investigated medication is not sure |
|  | In case of negative numbers, the medication causes additional side effects. |

Question 5

**What is the meaning of the size of confidence intervals?**

(Only one answer is correct)

|  | The size of the confidence interval does not say anything about the certainty of the results |
| --- | --- |
|  | A wider confidence indicates a few participants |
|  | Study results with wide confidence intervals stand for great trustworthiness |
|  | A wide confidence interval indicates many participants |

Question 6

**Which statement about confidence intervals is correct?**

| A narrow confidence interval …  *(Only one answer is correct)* | |
| --- | --- |
|  | … suggests that the benefit of a medication has been determined with considerable certainty |
|  | … is usually based on chance |
|  | … is worse than a wide confidence interval |
|  | … suggests that the benefit of a medication is uncertain |
